# Retracted: Subclinical Hypothyroidism and Its Association with Increased Cardiovascular Mortality

**DOI:** 10.1155/2018/9174206

**Published:** 2018-01-14

**Authors:** Cardiology Research and Practice

*Cardiology Research and Practice* has retracted the article titled “Subclinical Hypothyroidism and Its Association with Increased Cardiovascular Mortality” [1]. The article was previously published as “Hassan, A., Altamirano-Ufion, A., Zulfiqar, B., Boddu, P., Sub-Clinical Hypothyroidism and Its Association with Increased Cardiovascular Mortality: Call for Action. Cardiology Research, North America, 8, May 2017.” The authors appear to have inadvertently mixed up the two journals.
